# Disengagement and loss to follow-up in intravitreal injection clinics for neovascular age-related macular degeneration

**DOI:** 10.1038/s41433-023-02474-3

**Published:** 2023-03-14

**Authors:** Rebecca Jones, Irene M. Stratton, Peter H. Scanlon, Sofia Theodoropoulou

**Affiliations:** 1 Gloucestershire Retinal Research Group, Cheltenham General Hospital, Gloucestershire Hospitals NHS Foundation Trust, Cheltenham, GL53 7AN UK; 2 Nuffield Department of Orthopaedics, Rheumatology and Musculoskeletal Sciences, University of Oxford, Oxford, UK; 3 University of Southampton, Southampton, UK; 4 Nuffield Department of Clinical Neuroscience, University of Oxford, Oxford, UK; 5 University of Gloucestershire, Cheltenham, UK

**Keywords:** Macular degeneration, Biological therapy, Health services

## Abstract

**Background/Objectives:**

Timely assessment and treatment of patients with neovascular AMD (nAMD) are crucial to preservation of vision. Loss to follow up (LTFU) in these patients is a problem but this has not been systematically investigated.

**Subjects/Methods:**

A retrospective review of electronic medical records of patients with nAMD first treated with anti-VEGF therapy from 1st Jan 2014 to 31st Dec 2018, was conducted in January 2021. Any patient not seen for more than 12 months was classed as no longer attending.

**Results:**

Of the 1328 patients who attended between 2014 and 2018, 348 had failed to attend and were eligible for inclusion in this study. Reasons noted for discontinuation of care: discharged by clinician (33.3%), died (20.7%), moved to another unit outside of area (17.5%), stopped attending due to ill-health (13.5%), discharged due to failure to attend (5.6%) and patient choice to no longer attend (4.6%). There were 16 (4.6%) who did not receive any further appointments despite clinician request for follow-up. After 5 years, 50.5% of patients were no longer attending for treatment. Age was a factor in failure to attend, with 7 out of 12 patients aged >100 years no longer being followed up, compared to 1 out of 11 of 50–59 year-olds.

**Conclusions:**

When analysing visual outcomes in an AMD service it is important to characterise the patients who are lost to follow up. The outcomes for this group may be avoidably poor and understanding the factors influencing LTFU rate is crucial to addressing shortcomings in a hospital AMD service.

## Introduction

Age-related macular degeneration (AMD) is the second most common cause of visual impairment in the developed world. The prevalence of cases is increasing due to the aging population [1]. In the UK, cases of neovascular AMD are predicted to rise by 59% from 2015 to 2035 [2]. Landmark randomised controlled trials have demonstrated a significant benefit to visual outcomes with regular treatment with anti-Vascular Endothelial Growth Factor (VEGF) intravitreal injections [3, 4]. The majority of patients commenced on anti-VEGF therapy require this long-term with frequent injections [5]. Provision of therapy to this growing population of patients poses a burden on hospital eye services.

In the UK, the cost of anti-VEGF treatment is covered by the National Health Service and is free-of-charge for the patient. Our tertiary referral centre follows the Treat and Extend regimen for treatment, with patients receiving intravitreal injections at intervals based on their disease activity [6]. If the Optical Coherence Tomography (OCT) appearance of the choroidal neovascular membrane remains stable without signs of activity, the interval between injection is extended. Real-world studies have shown that it is possible to demonstrate visual outcomes equivalent to those in clinical trials when patients are followed-up and treated according to protocol [7]. It is reasonable to assume that by discontinuing care, visual outcomes are likely to be worse and it is, therefore, important to recognise any barriers to continuation of treatment that could be prevented.

This study aimed to identify patients with neovascular AMD (nAMD) who were no longer attending regular hospital follow-up and the rate at which they dropped out of treatment. Our aim was to investigate factors influencing discontinuation of anti-VEGF therapy.

## Methods

A retrospective electronic case notes review was undertaken of patients who received an initial intravitreal injection of anti-VEGF (aflibercept, ranibizumab or bevacizumab) during the period 1st January 2014 to 31st December 2018 at Gloucestershire Hospitals NHS Foundation Trust. These data were taken from electronic medical records (EMR) kept on Medisoft or Intersystems TrakCare. Those over 50 years of age with a diagnosis of neovascular AMD and who received an initial injection of aflibercept, ranibizumab or bevacizumab in either their first or second eye during this time period were included in analysis.

The following data were recorded for each patient: age at time of data collection (or at death), gender, first and last visit dates, LogMAR best-corrected visual acuity (BCVA) at baseline and at last follow-up visit in the treatment and fellow eye (or both eyes if on bilateral treatment), number of injections in treatment eye, whether on regular treatment or post-treatment observation, and presence of active or inactive disease in the fellow eye.

Patients who did not have a follow up appointment for greater than 12 months were recorded as no longer attending, and therefore included in our study. For these patients, EMR entries recording reasons for ceasing to attend were noted. This data had been accurately recorded over the 5-year period by the AMD coordinators with entries made onto the electronic health record if a patient phoned up or cancelled an appointment and we have reported the data that they recorded. If patients failed to attend a minimum of three booked appointments without contacting to rearrange, this led to an automatic discharge from follow up. The reasons provided for discontinuation of treatment included: follow-up cancelled by patient, patient deceased, discharged by clinician, multiple failures to attend leading to automatic discharge, ill-health, or transfer to another unit. Patients discharged were divided into two groups: those in whom further treatment was felt to be futile (below treatment criteria = BTC), either due to failure to improve or progressive decline in visual acuity despite treatment, or those discharged after a period of disease inactivity on OCT.

## Results

A total of 1328 patient received an initial anti-VEGF injection for nAMD in the study period, January 2014 to December 2018. The mean age was 84 years (range: 53–103 years) at death or last visit. There were 348 who had not had a follow-up appointment after 1 year from last appointment, and were therefore assumed to have discontinued care: 223 women (64.1%) and 125 men (35.9%). The mean duration of follow-up for these patients was 26.3 months (±2.1), with on average 6.8 (±5.0) injections received.

In 116 patients (33.3%), the primary reason for stopping follow-up was discharge from hospital eye services. All of these patients had ceased anti-VEGF therapy. They had inactive disease with stable visual acuity and OCT appearance (*n* = 71), or their BCVA was consistently below treatment criteria (LogMAR 1.20) and further anti-VEGF injections were thought to be futile (*n* = 45). Table 1 shows the pre- and post-treatment visual acuities in these two groups of discharged patients, and the number of injections prior to discharge. Patients who were discharged due to consistent disease inactivity showed a mean improvement of BCVA of LogMAR 0.12 (range: letter score loss of 50 letters to gain of 59 letters; LogMAR loss of 1.0 to gain of 1.18) from baseline to final visit (*p* = 0.006, paired sample *t*-test). The average age in this group was 87 years (range: 62–103 years), and 61.2% were women.

**Table 1:** Demographics of patients who were discharged by their lead clinician from further follow up, and their outcomes of treatment, divided into those who were discharged due to visual acuity worse than treatment criteria, and those who had stable inactive disease, not requiring further treatment.

	Discharged from follow up	
Total number of patients	116	
Mean Age (years), range	87.15, 62–103	
Gender:		
Male, number/total, %	45/116, 38.2%	
Female, number/total, %	71/116, 61.2%	
	**Stopped because below treatment criteria**	**Stable inactive disease**
LogMAR visual acuity at initiation of treatment, mean	0.96	0.61
LogMAR visual acuity at last recorded appointment, mean	1.68	0.49
Number/total whose visual acuity deteriorated over the treatment period, percentage	37/45, 83%	13/71, 18%
Months in treatment, mean	32.58	32.22
Number of anti-VEGF injections, mean	7.02	6.42

Of those no longer attending follow up, 72 (20.7%) had died. A further 47 (13.5%) patients were unable to attend further follow up due to ill-health. Sixty-one patients (17.5%) moved out of the Gloucestershire area to seek further follow-up in another NHS Trust.

The remaining LTFU patients whose anti-VEGF therapy abruptly stopped were those who declined any further follow up (*n* = 16, 4.6%), those who did not attend on more than three occasions and were automatically discharged (*n* = 20, 5.8%), and finally those who did not receive any further appointments despite clinician request for follow-up (*n* = 16, 4.6%). Further detail about these patients is provided in Table 2. Of those who declined further treatment, 3 patients found injections too painful, 2 patients wanted no further treatment following endophthalmitis, and 2 patients reported difficulty arranging transport to their appointments.

**Table 2:** Details on characteristics of the 52 patients, who were classed as LTFU due to patient choice to decline follow up, multiple missed appointments leading to discharged, or administrative error leading to no further appointments being sent.

	Cancelled by patient	Multiple missed appointments	No appointments sent
Total number of patients	16	20	16
Mean Age (years), range	89.75, 70–99	88, 63–101	88.5, 73–99
LogMAR visual acuity at initiation of treatment, mean	0.65	0.54	1.12
LogMAR visual acuity at last recorded appointment, mean	0.8	0.55	1.64
Months in treatment, mean	16.75	19.90	28.63
Number / total on active treatment protocol, percentage	13/16, 81.25%	13/20, 65%	1/16, 6.25%
Visual acuity in fellow eye, mean	0.46	0.5	0.81
Number / total on active treatment for nAMD in both eyes, percentage	1/16, 6.25%	3/20, 15.0%	0%
Number of injections, mean	7	8.25	6.19

**Fig. 1:**
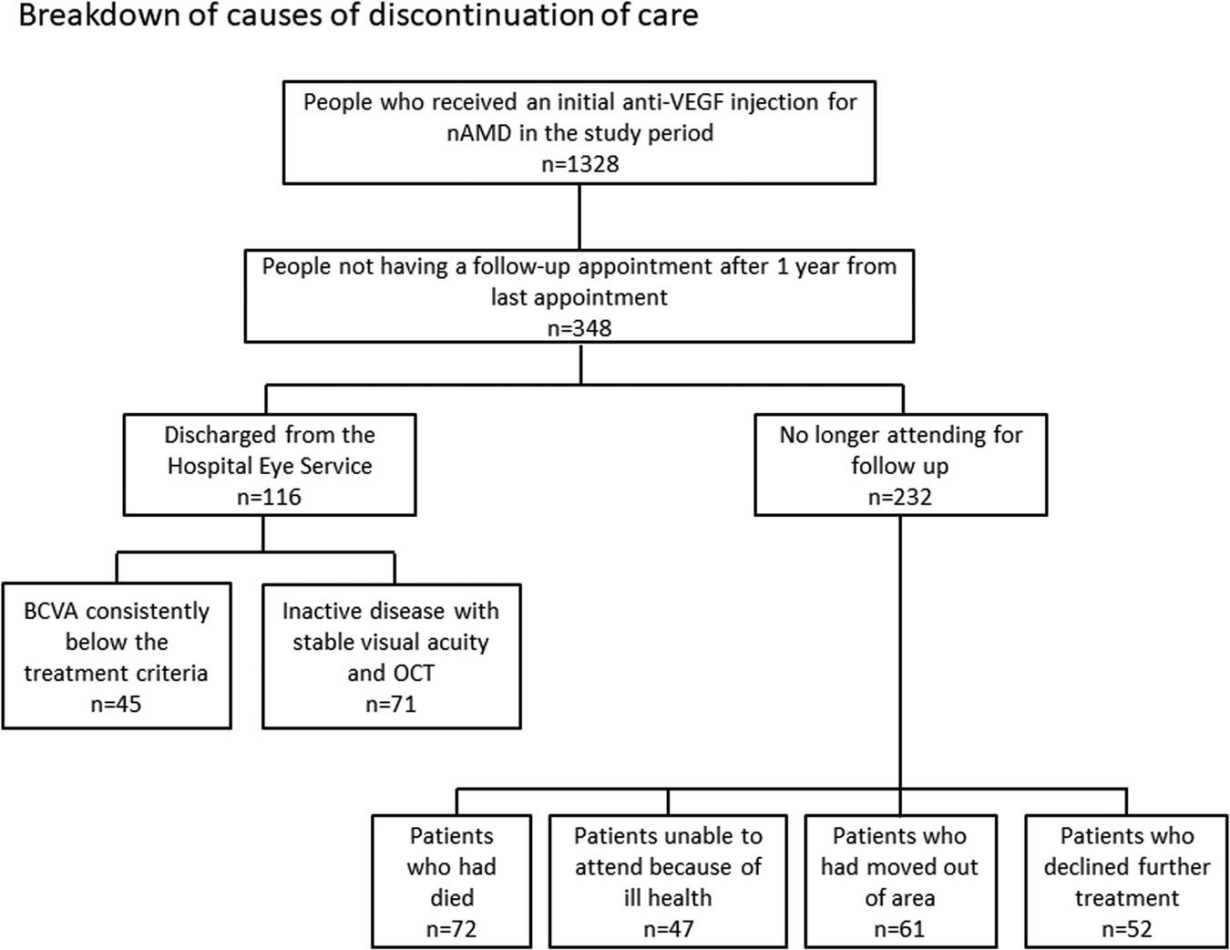
Flow diagram. Reasons for discontinuation of care and the number of patients in each subgroup.

**Fig. 2:**
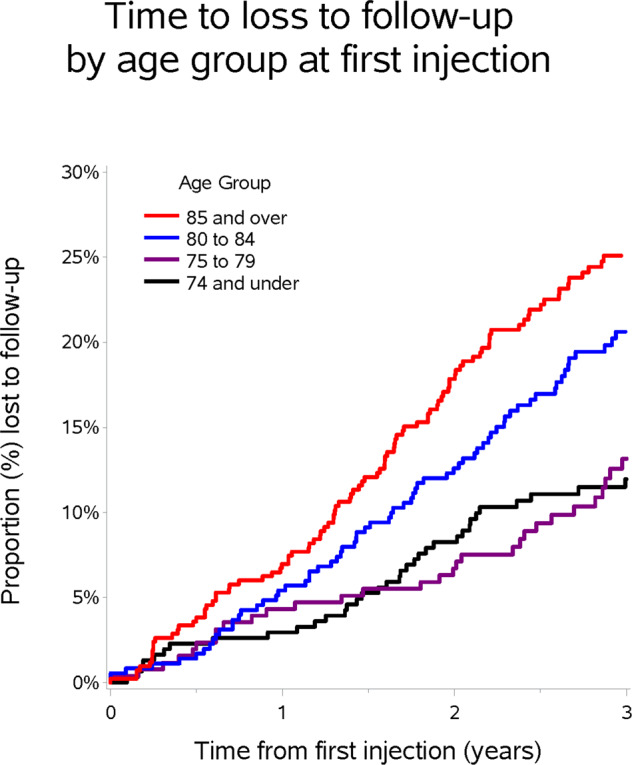
Kaplan-Meier Curve. Demonstrating time to loss to follow-up by age group at first injection.

In our cohort, after 5 years, half of patients had dropped out of treatment or died. Figure 1 demonstrates a breakdown of causes of discontinuation of care. Age was also identified as an independent factor in failure to attend, with 58% of patients aged >100 years no longer being followed up, compared to 9.1% of 50–59 year-olds (see Fig. 2 for drop-out rate by age). The average age of those LTFU was 88 years and those remaining under follow up were 83 years.

## Discussion

This study was instigated in response to the 2014 HQIP commissioned National Ophthalmology Audit published in 2017 in which loss to follow up data were provided [8]. There was no information available in the audit on the reasons for this. Studies have shown benefit from intravitreal anti-VEGF therapy given at regular intervals, for the treatment of neovascular AMD. It is essential to recognise the real-world outcomes and minimise non-compliance with care to try and bring the non-clinical trial setting closer in line with the RCT participants.

The rate of drop-out after 5 years in this cohort was 50%. This is not dissimilar to the rates reported by Boulanger-Scemama (57% at 5 years) and Gillies (53% at 5 years) and comparable to those reported by Pushpoth in another UK-based study (49% at 4 years) and by Falk (47% at 4 years) [9–12]. Vaze reported a lower rate of discontinuation of treatment, of 42% treatment discontinuation over 6 years [13]. Studies with a shorter follow up period had a comparably higher rate of LTFU, with Oishi reporting a 33% rate after 6 months of treatment, not including those who were discharged following remission in their disease activity [14]. In our cohort, only 6% of patients failed to attend 1 year into treatment. Vaze reported the reasons for treatment discontinuation in their cohort. 10.5% of patients declined treatment and 14.5% were discharged [13]. In comparison, in our cohort, a larger proportion were discharged (33.3%) and fewer patients cancelled follow-up (4.60%). When Rosenblatt et al looked at rate of treatment discontinuation in major anti-VEGF injection trials, they found a mean rate of drop-out of 12.44% in the trial populations, in studies ranging from 12 weeks to 3 years duration [15].

We found that few patients cancelled appointments due to anxiety around injections or found injections too painful to continue. Of our cohort, this was only the case for four patients, who were all LTFU in the first 2 years of treatment. These patients had six injections before cancelling follow up, demonstrating a window of opportunity to counsel patients or discuss options for anaesthesia to prevent drop out.

Comparable studies have shown that issues with transport are a major burden to patients who have to attend regularly [16]. In Droege’s questionnaire of patients on ranibizumab for neovascular AMD, 46% reported that “Travel to/from the hospital was generally a problem” [17]. However, in our cohort, only two patients reported difficulty in transport as a reason for no longer attending. This proves reassuring that transportation difficulties are not a barrier to attendance for treatment. The population in Gloucestershire access treatment at two hospitals, covering both urban and rural areas, with an average population density of 4 people per hectare. It is therefore promising that this did not have a major impact on patients’ attendance to follow up.

It is of concern that 16 people were not sent a follow up appointment. Of these 16 patients, 15 were not receiving regular treatment because the choroidal neovascular membrane had stabilised. It is possible that these patients had therefore been discharged, but without the decision being formally documented in their medical records. The final patient who was not sent an appointment had initially cancelled due to travelling and later died. These patients who are lost to follow up due to administrative errors, rather than clinician or patient choice, are extremely important to identify to prevent avoidable harm. As reported by Davis, vulnerable patients are at higher risk of harm from LTFU, and AMD patients are in this category, as many are elderly or visually impaired [18].

The limitations of this study include its retrospective design, relying on the accuracy of clinical documentation. Using this method of patient identification for LTFU may also miss some patients, as some may not have been seen for more than 12 months and then been re-referred to our service, and, therefore, not been included in this dataset. Greater than 12 months since last visit was deemed to be a sufficient time period to class as LTFU, as it was felt unlikely that patients would have an interval greater than one year between appointments. This is also the consensus from other authors [16, 19]. It is possible that patients that have been LTFU but not yet met 12 months since their last appointment have been missed from this study. We did not assess the clinician recommended follow-up interval, and whether this had been met, to evaluate true dropout.

This study provides reassuring evidence that when the decision is made to permanently stop treatment in nAMD, it is a decision that is usually made by the lead clinician rather than the patient due to pain, anxiety, transport issues or unsatisfaction with visual outcomes. This appreciation of the causes for loss to follow up, and those at higher risk, allows us to better personalise services in the future to improve patient outcomes. Further analyses of the reasons for LTFU from larger datasets, such as the National Ophthalmology AMD dataset, will allow greater planning of service provision.

## Summary

### What was known before

- In 2014 the Health Quality Improvement Programme commissioned the Royal College of Ophthalmologists to undertake a feasibility audit in the treatment of “wet” Neovascular Age Related Macular Degeneration.
- In the feasibility audit data were not available to explain why patients were lost to follow-up and only six of 32 centres had any patients reported as having died.

### What this study adds

- This study provides a detailed account of the reasons for loss to follow up, which should provide a benchmark for services to compare their loss to follow up results with in the newly commissioned National Audit of treatment of “wet” Neovascular Age Related Macular Degeneration.

## Data Availability

The datasets generated during and/or analysed during the current study are available from the corresponding author on reasonable request.
