# Correction to: Disengagement and loss to follow-up in intravitreal injection clinics for neovascular age-related macular degeneration

**DOI:** 10.1038/s41433-023-02589-7

**Published:** 2023-06-30

**Authors:** Rebecca Jones, Irene M. Stratton, Peter H. Scanlon, Sofia Theodoropoulou

**Affiliations:** 1 Gloucestershire Retinal Research Group, Cheltenham General Hospital, Gloucestershire Hospitals NHS Foundation Trust, Cheltenham, GL53 7AN UK; 2 Nuffield Department of Orthopaedics, Rheumatology and Musculoskeletal Sciences, University of Oxford, Oxford, UK; 3 University of Southampton, Southampton, UK; 4 Nuffield Department of Clinical Neuroscience, University of Oxford, Oxford, UK; 5 University of Gloucestershire, Cheltenham, UK

**Keywords:** Macular degeneration, Biological therapy, Health services

Correction to: *Eye* 10.1038/s41433-023-02474-3, published online 14 March 2023

The article “Disengagement and loss to follow-up in intravitreal injection clinics for neovascular age-related macular degeneration”, written by Rebecca Jones, Irene M. Stratton, Peter H. Scanlon and Sofia Theodoropoulou, was originally published electronically on the publisher’s internet portal on 14 March 2023 without open access. With the author(s)’ decision to opt for Open Choice the copyright of the article changed on 22 April 2023 to © The Author(s) and the article is forthwith distributed under a Creative Commons Attribution 4.0 International License, which permits use, sharing, adaptation, distribution and reproduction in any medium or format, as long as you give appropriate credit to the original author(s) and the source, provide a link to the Creative Commons licence, and indicate if changes were made. The images or other third party material in this article are included in the article’s Creative Commons licence, unless indicated otherwise in a credit line to the material. If material is not included in the article’s Creative Commons licence and your intended use is not permitted by statutory regulation or exceeds the permitted use, you will need to obtain permission directly from the copyright holder.To view a copy of this licence, visit http://creativecommons.org/licenses/by/4.0/.

